# Delayed Soft Tissue Reconstruction with a Horizontal Rectus Abdominis Musculocutaneous Flap following Hip Exarticulation

**DOI:** 10.1155/2013/670304

**Published:** 2013-08-19

**Authors:** Jes Christian Rødgaard, Troels Michael Tei

**Affiliations:** Department of Surgery, Section of Plastic and Reconstructive Surgery, Sydvestjysk Sygehus, Finsensgade 35, 6700 Esbjerg, Denmark

## Abstract

Coverage of large soft tissue defects at the hip region constitutes a challenge for plastic surgeons. We report the case of a 43-year-old female with necrotizing fasciitis of the right thigh, necessitating hip exarticulation and substantial debridement of necrotic tissue. An ipsilateral horizontal rectus abdominis myocutaneous (HRAM) flap was used to cover the defect. The reconstruction was carried out after the attempt of local tissue rearrangement. In light of the successful outcome, we propose that this flap be considered in the future planning of soft tissue reconstruction at the hip region.

## 1. Introduction

**Figure 1:**
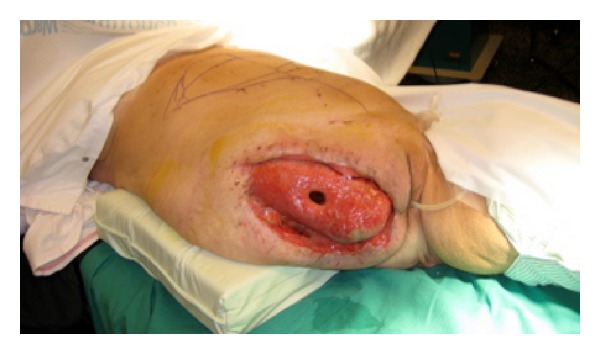
Soft tissue defect 3 months after hip exarticulation. Acetabulum is exposed centrally.

Delayed soft tissue reconstruction after hip exarticulation is a surgical challenge. We present a case in which a distal pedicled ipsilateral horizontal rectus abdominis musculocutaneous (HRAM) flap was used to cover a large tissue defect following hip exarticulation due to necrotizing fasciitis (Figure 1). Instead of using a conventional vertical or transverse rectus abdominis musculocutaneous (VRAM/TRAM) flap, we used a rectus flap with a supraumbilical horizontal skin island. This has, to our knowledge, not previously been described.

## 2. Case History

**Figure 2:**
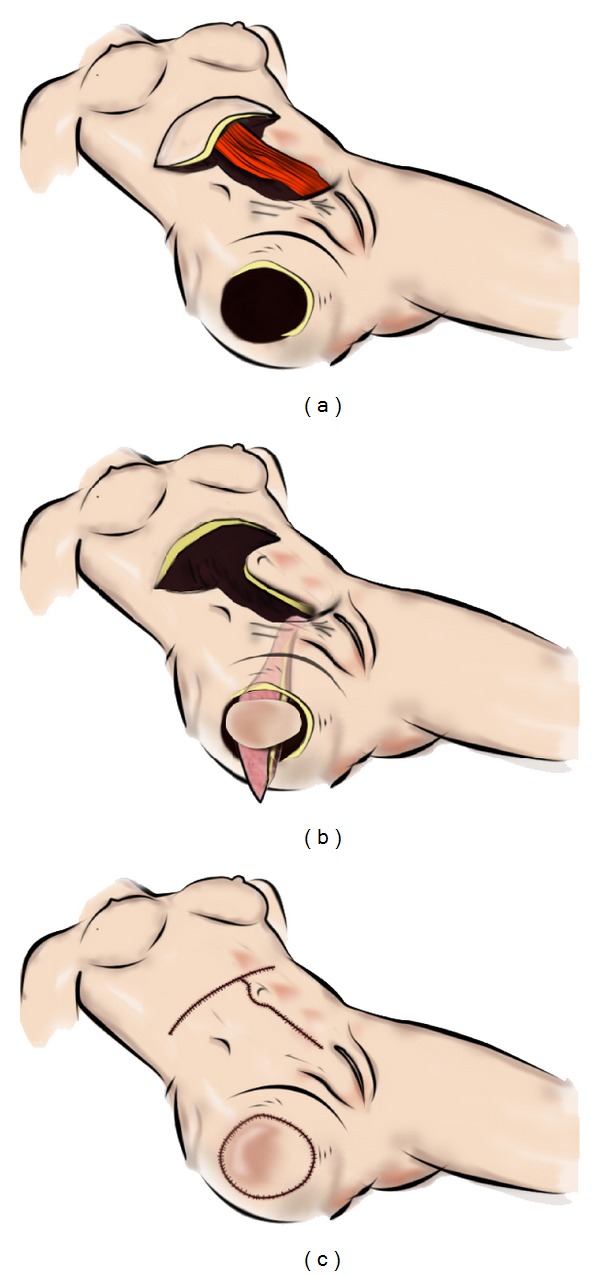
Design of ipsilateral distal pedicled horizontal rectus abdominis musculocutaneous flap.

A 43-year-old female with a history of drug addiction was admitted to hospital in 2007 due to pain in the right leg, with swelling and pyrexia. Initial examination revealed swelling and erythema with purpura over the right hip and thigh region. Urgent contrast enhanced computed tomography (CT) scan revealed marked inflammatory stranding and low attenuation with suspicion of necrosis. The patient was immediately admitted to the surgical intensive care unit where a fasciotomy was performed. The necrosis was extensive, and exarticulation of the right hip was necessary. Substantial surgical debridement was made, and most of the gluteus musculature was removed. Primary closure of the soft tissue defect was not achievable, and vacuum-assisted closure therapy was applied the first month. Microbiology of the wound biopsy revealed group A streptococcus, and the patient was treated with Vancomycin and Metronidazol. Repeated wound-revisions and the use of vessel loops for secondary wound closure were carried out unsuccessfully. Three months after the initial operation, it was decided to perform a reconstructive procedure involving a pedicled rectus abdominis musculocutaneous flap (Figure 2).

### 2.1. Operative Technique

**Figure 3:**
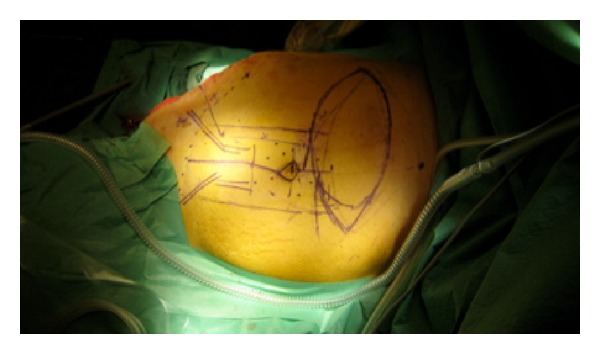
Preoperative markings.

**Figure 4:**
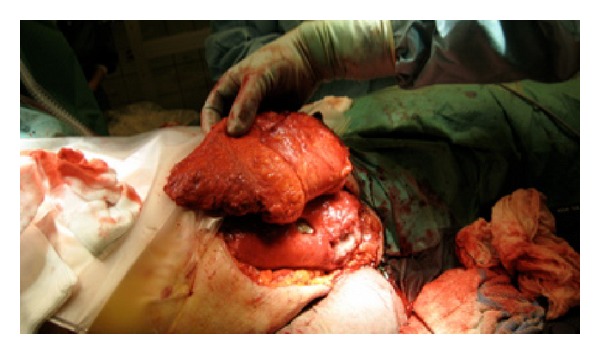
Intraoperative photograph with the flap tunneled subcutaneously.

**Figure 5:**
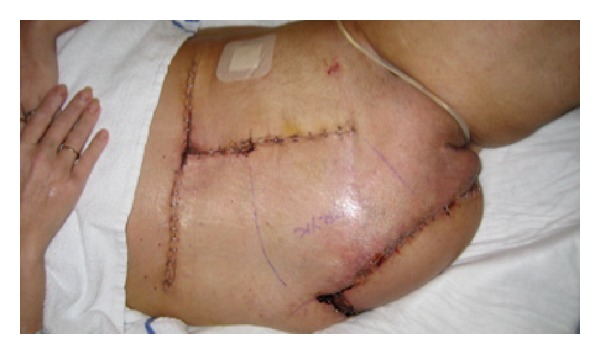
Postoperative photograph.

The technique of distal placement of VRAM flaps has been described in detail previously [1]. This flap has a horizontal skin island design. The skin island was designed to be supraumbilical, 2/3 parts situated in zone 1 and 3 according to Taylor et al. axiome concept, and 1/3-part on zone 4 (Figure 3). When harvesting the flap, attention was taken to include infraumbilical perforators within 5 cm from umbilicus to ensure sufficient flap vascularization. The flap was separated from the posterior sheet of the rectus muscle, and fascia-sparring technique of the anterior sheet was carried out. Subsequently the flap was isolated to the level of the deep distal epigastric vessels. The muscle was transected caudal to the rib curvature, and the flap was tunneled subcutaneous to the defect, measuring 25 cm × 35 cm (Figure 4). The tuber ischium prominence was resected to facilitate the inset of the flap. Furthermore the reminiscent from the acetabula cavity was obliterated by gentamycin-enriched cement, performed in an orthopedic multidisciplinary cooperation. A synthetic mesh was used to reconstruct the integrity of the abdominal facial wall to ensure tension-free closure of the rectus-sheet. Single suction tube drainage was positioned under the flap at the donor site (Figure 5). An abdominal suspension belt was applied for 3 months after the operation.

## 3. Discussion

The distally based rectus abdominis musculocutaneous flap offers several advantages, including a reliable pedicle, a large arc of rotation and a substantial bulk of tissue to cover large tissue defects. Tension free closure of the abdominal wall defect is critical to prevent donor site morbidity, including bulging and incisional hernia. The indication to make a free rectus abdominis flap is rarely given, since alternative free flaps offer an equal size of skin paddle and less donor-site morbidity.

If the skin island is designed above umbilicus, the flap is claimed to be more robust [2] in comparison with a conventional infraumbilical skin island. The flap is supplied anatomically by the deep inferior and superior epigastric artery (DSEA respective DIEA), which anastomoses above umbilicus, and further vascular anastomoses exist with the ipsilateral intercostal vessels. This flap encounters the concept described decades ago by Taylor and Palmer: “The vascular territories (angiosomes) of the body” [3], and in recent studies confirmed by Saint-Cyr et al. [4] and Rozen et al. [5], describing the concept in which safe clinical cutaneous perforators extend beyond their anatomical territory to include the next cutaneous perforator, typically situated radical in any direction. The knowledge of this concept is essential when conducting perforator-based flaps or in this case, a musculocutaneous flap covering a 25 cm × 35 cm skin defect.

In cases of covering large soft tissue defects following previous hip exarticulation, a variety of flaps can also be considered. The latissimus dorsi muscle is mainly used as a free flap, but surgeons should also consider the pedicled reverse muscle flap. The reversed flap has a limited arc of rotation, but by trimming the musculocutaneous flap to a muscle flap, the area reached can be extended by “turning over” the flap [6]. Eventually, the area covered can reach the superior anterior iliac spine, however not sufficiently to cover the defect in our patient. In certain cases, a pedicled lumbar perforator flap is another alternative, mainly in covering defects in the posterior hip region or in a combination with other flaps available [7]. The pedicled external oblique musculocutaneous flap serves as an alternative when workhorse flaps are unavailable [8]. The underlying muscle, the internal oblique, is also an option. Both muscles are sufficiently bulky to cover large defects but possess the disadvantage of undermining the integrity of the abdominal wall, with the risk of hernia formation. Gluteus muscle transposition is widely used for pelvic defects, but in our case most of the musculature was debrided as a result of necrosis, which precluded its use.

Immediate reconstruction offers additional opportunities. Harvesting of a pedicled thigh fillet flap or a free lower limb fillet is based on “the spare part” concept and has the advantage of eliminating donor site morbidity [9]. For more superficial defects, the fasciocutaneous anterolateral thigh flap can be used [10].

The challenge for the reconstructive surgeon is to select which flap would be most appropriate for each individual patient. Since each method of reconstruction has inherent advantages and disadvantages, the choice of method should be made on a case-by-case basis. A distal HRAM flap provides reliable soft tissue coverage of extensive tissue defects following hip exarticulation or other aetiology for defect in hip-near region, with an acceptable outcome.
